# Elevated long noncoding RNA MALAT-1 expression is predictive of poor prognosis in patients with breast cancer: a meta-analysis

**DOI:** 10.1042/BSR20200215

**Published:** 2020-08-11

**Authors:** Yanyan Wang, Yujie Zhang, Kaimin Hu, Jili Qiu, Yue Hu, Meiqi Zhou, Suzhan Zhang

**Affiliations:** 1 Department of Oncology Surgery, The Second Affiliated Hospital, Zhejiang University School of Medicine, 88 Jiefang Road, Hangzhou 310009, China; 2 Department of Orthopedic Surgery, The Second Affiliated Hospital, Zhejiang University School of Medicine, 88 Jiefang Road, Hangzhou 310009, China

**Keywords:** breast cancers, Long noncoding RNA, MALAT-1, prognosis

## Abstract

Accumulating evidence indicates that aberrant regulation of metastasis-associated lung adenocarcinoma transcript 1 (MALAT-1), a long noncoding RNA (lncRNA), plays a vital role in tumorigenesis. However, its association with breast cancer has not been systematically evaluated. In the current study, a meta-analysis was conducted to clarify the association between MALAT-1 and the prognosis and clinicopathological features of breast cancer. Relevant literature published in several databases was searched. Hazard ratio (HR) and odds ratio (OR) with 95% confidence interval (CI) were calculated to evaluate the effect of MALAT-1 expression on the survival outcomes and clinicopathological features of breast cancer. A total of 12 studies involving 4106 patients were identified. Pooled HR demonstrated that elevated MALAT-1 expression significantly predicted unfavorable overall survival (HR = 2.06, 95% CI: 1.66–2.56, *P*<0.0001) in patients with breast cancer. Subgroup analysis stratified by cancer type, sample size, and method of variance analysis also showed statistically significant associations. Additionally, the HR of patients with up-regulated MALAT-1 expression concerning disease-free survival (DFS), recurrence-free survival (RFS), and disease-specific survival (DSS) was 1.91 (95% CI: 1.53–2.39, *P*<0.0001). Further, elevated MALAT-1 expression was positively correlated with the progesterone receptor (PR) status (OR = 1.47, 95% CI: 1.18–1.82). Thus, MALAT-1 is a promising biomarker for predicting survival outcomes in patients with breast cancer.

## Introduction

Breast cancer is the most common malignancy affecting females, and although substantial progress in terms of its management has been made, it still remains the major cause of cancer-related deaths among females worldwide due to recurrence and metastasis [1,2]. Breast cancer is a heterogeneous disease that exhibits considerable variability in its prognostic patterns and treatment response [3,4]. Molecular biomarkers have been identified as promising candidates and may not only predict the biological behavior and clinical outcome but also contribute to the improvement of treatment protocols [5–7].

Long noncoding RNAs (lncRNAs), with a length of more than 200 nucleotides, do not encode proteins and were previously regarded as ‘transcriptional noise’ or ‘junk RNA’; however, a growing number of studies have indicated that lncRNAs are key regulators that influence various biological processes [8,9]. LncRNAs have been found to be consistently dysregulated and closely related to carcinogenesis and tumor progression in many cancers including liver, pancreas, colon, lung, prostate, and breast cancers [10]. In particular, several lncRNAs are considered to be promising prognostic markers in cancer [11,12].

Metastasis-associated lung adenocarcinoma transcript 1 (MALAT-1), approximately 8000 nucleotides in length, is one of the most conserved lncRNAs and is extremely abundant and ubiquitously expressed in different tissues [13]. MALAT-1 was initially recognized as a metastasis-associated gene that might predict the potential metastasis of non-small-cell lung cancer [14]. There is accumulating evidence that dysregulated MALAT-1 is closely associated with the development of various tumors. For example, MALAT-1 can promote cell invasion and migration by serving as a competitive endogenous RNA (ceRNA) to attenuate the function of miR-200c-3p/ZEB1 in pancreatic ductal adenocarcinoma [15]. MALAT-1 has also been shown to induce epithelial–mesenchymal transition through the miR-204/ZEB2 axis in breast cancer [16]. A previous meta-analysis has shown that the up-regulation of MALAT-1 is significantly correlated with the prognosis and metastasis of cancers [17]. Nevertheless, the prognostic role of MALAT-1 in breast cancer is poorly characterized, with no systematic evaluation conducted to date [18,19]. Therefore, we conducted a systematic review to investigate the associations of MALAT-1 with clinicopathological features and survival of patients with breast cancer.

## Materials and methods

### Identification

We carefully searched international databases (Web of Science, PubMed, and Embase) to identify studies referring to MALAT-1 in breast cancer. We used the following search terms or key words ‘MALAT-1 OR MALAT1 OR metastasis-associated lung adenocarcinoma transcript 1’ and ‘breast or mammary’ and ‘tumor OR cancer OR carcinoma OR neoplasm.’ The last update of our research was conducted on 22 January 2020.

### Inclusion and exclusion criteria

Studies fulfilling the following criteria were regarded as eligible studies: (1) the expression of MALAT-1 was measured in either tumor tissue or serum; (2) patients in the study were diagnosed with breast cancer; (3) the relationship between MALAT-1 expression and clinicopathological features or prognosis in breast cancer patients was investigated; and (4) odds ratios (ORs) or hazard ratios (HRs) and 95% confidence interval (CI) were recorded or sufficient published information was provided to calculate them. Studies meeting any of the following criteria were excluded: (1) important information including clinicopathological parameters and survival outcomes was not available; (2) studies were in the form of reviews, letters, comments, or conference abstracts; (3) studies were published in a language other than English; and (4) studies that reprocessed data from public databases.

### Quality assessment and data extraction

Two investigators (Y.y.W. and Y.j.Z.) independently assessed all potentially eligible studies based on the Newcastle–Ottawa Scale (NOS) [20]. Articles with NOS ≥ 6 were regarded as high-quality studies. Any discrepancy was resolved by discussion. We extracted the following required information: (1) first author and publication year; (2) patient characteristics (country, ethnicity, sample size, type of specimen, histological grade, stage of disease, hormone receptor including progesterone receptor (PR) and estrogen receptor (ER) status, human epidermal growth factor receptor 2 (HER2) status, lymphoid node status, and follow-up); (3) detection methods of MALAT-1 and cut-off value; and (4) HRs and the corresponding 95% CI of up-regulated MALAT-1 for overall survival (OS), metastasis-free survival (MFS), disease-specific survival (DSS), disease-free survival (DFS), or recurrence-free survival (RFS). We also utilized Engauge Digitizer (version 4.1) to estimate the HRs based on the graphical survival plots when HRs were not directly available in the study [21]. We also corresponded with relevant original authors for additional data required for meta-analysis.

### Breast cancer patient cohort analysis based on public datasets

The gene expression profile of GSE19615 containing chemotherapy-treated breast cancer samples was obtained from the Gene Expression Omnibus (GEO) database (https://www.ncbi.nlm.nih.gov/geo/). The genome expression data of breast cancer samples from The Cancer Genome Atlas (TCGA) database (https://portal.gdc.cancer.gov/) were also downloaded. MALAT-1 expression levels were compared between the two groups according to whether the included patients received chemotherapy. Kaplan–Meier survival analysis was then employed to compare the survival outcomes between groups. Given the vital roles of immune infiltration in the tumorigenesis and metastasis, we applied the CIBERSORT algorithm to determine the association between MALAT-1 and infiltrating immune cells from gene expression profiles. The correlation was evaluated using the Pearson’s correlation coefficient; an estimated *P*-value of <0.05 was considered statistically significant.

### Statistical analysis

HRs and their associated 95% CIs for OS, MFS, DFS, DSS, and RFS were used to evaluate the prognostic effect of MALAT-1 in breast cancer. An HR > 1 and 95% CI not including 1 suggested a significant association between poor prognosis and elevated MALAT-1 expression. Cochran’s Q test and *P*-values were applied to find the heterogeneity among studies. If the calculation suggested significant heterogeneity (*I^2^* ≥ 50% and/or *P*≤0.05), the random-effects model was utilized. Otherwise, the fixed-effects model was more appropriate. ORs and their corresponding 95% CIs were calculated to determine the association of MALAT-1 with the clinicopathological variables of breast cancer. We used a sensitivity analysis to evaluate the stability of the results. The possibility of publication bias was also tested through Egger’s test and Begg’s funnel plot. All statistical analyses were performed using the Stata 15.0 software.

## Results

### Summary of the included studies

**Figure 1:**
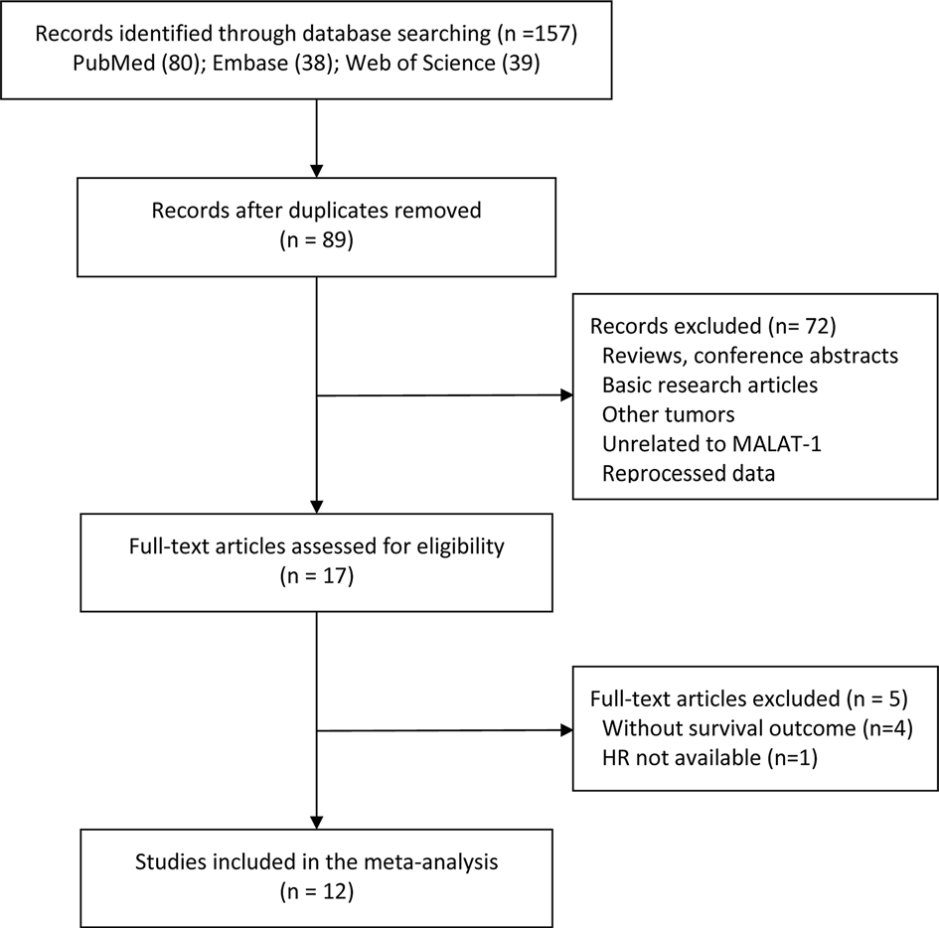
Flow chart depicting the literature selection procedure in this meta-analysis

Based on the Web of Science, PubMed, and Embase databases, we initially retrieved 157 papers, 68 of which were excluded due to duplication. After careful screening of the titles and abstracts, 17 potentially eligible articles were reviewed in detail. Due to the lack of key information regarding survival outcomes, 5 of the 17 articles were subsequently excluded; hence, we finally included a total of 12 articles in the meta-analysis [16,18,19,22–30]. Figure 1 summarizes the details of the literature selection procedure.

A total of 4186 patients from China, France, and the United States were investigated, with a minimum and maximum sample size of 43 and 1992 patients, respectively. MALAT-1 expression levels were assessed using reverse transcriptase polymerase chain reaction (RT-PCR) in all studies, with 11 studies employing cancerous tissue and 1 using serum specimens [26]. The cut-off estimates for MALAT-1 expression, including the median, fold change, and expression level, were inconsistent among these studies. Elevated MALAT-1 expression levels in breast cancer were reported in most studies, except in one study that reported the down-regulation of MALAT-1 [18]. Among the different cancer subtypes, Jadaliha et al. found that MALAT-1 expression was consistently higher in ER-positive patients than in those with the triple-negative breast cancer (TNBC) subtype [24]. They also suggested that TNBC subtype cells express relatively lower levels of MALAT-1 than other subtypes of breast cancer cells. There were two studies which focused only on TNBC [22,28]. Of these, Huang et al. divided all patients into groups based on receptor status, with ER-positive or -negative groups [23], and Jadaliha et al. did not report a negative result in all patients but mentioned the survival outcomes in the lymph node-negative TNBC and HER2-positive breast cancer subsets [24]. Eight of all included studies described the clinicopathological features of breast cancer [16,18,19,22,23,25,26,30]. Of the 12 articles, 2 studies only described the survival outcomes of breast cancer, without providing necessary data to obtain HR [18,25]. HR was reported directly in four studies [19,23,24,27] and estimated indirectly for the remaining six studies [16,22,26,28–30]. Table 1 shows the detailed information of the included studies.

**Table 1:** Main characteristics of the studies included in the present meta-analysis

Author’s name	Year	Country	Number	Type	Cut-off	Follow-up (months)	Sample	Detection method	Expression level	HR	Survival outcomes	Multivariate analysis	NOS score
Jin [22]	2015	China	139	TNBC	median	55	tissue	RT-PCR	↑	SC	OS	No	7
Xu [18]	2015	China	135	Mixed	NR	NR	tissue	RT-PCR	↓	NR	NR	No	6
Huang [23]	2016	China	204	ER+ and ER−	75% expression	65	tissue	RT-PCR	↑	Reported	RFS	No	8
Jadaliha [24]	2016	U.S.A.	1992	TNBC and Her2+	NR	NR	tissue	RT-PCR	↑	Reported	DSS	No	6
Meseure [25]	2016	France	446	Mixed	3.02-fold	NR	tissue	RT-PCR	↑	NR	MFS	No	7
Miao [26]	2016	China	78	Mixed	median	60	tissue	RT-PCR	↑	SC	DFS	No	8
Wang [16]	2017	China	118	Mixed	NR	50	tissue	RT-PCR	↑	SC	OS	No	7
Zhang [27]	2017	China	178	Mixed	3.48	NR	serum	RT-PCR	↑	Reported	OS	Yes	7
Zuo [28]	2017	China	43	TNBC	median	60	tissue	RT-PCR	↑	SC	OS	No	7
Li [29]	2018	China	258	Mixed	NR	23	tissue	RT-PCR	↑	SC	RFS	No	7
Wang [19]	2018	China	509	Mixed	median	82.8	tissue	RT-PCR	↑	Reported	OS/RFS	Yes	7
Zhang [30]	2018	China	86	Mixed	median	NR	tissue	RT-PCR	↑	SC	OS	No	6

Abbreviations: ER+, ER positive; ER−, ER negative; Her2+, Her2 positive; NM, not mentioned; NR, not reported; qRT-PCR, quantitative reverse transcription-polymerase chain reaction; SC, survival curve.

### Association between MALAT-1 expression and OS

**Figure 2:**
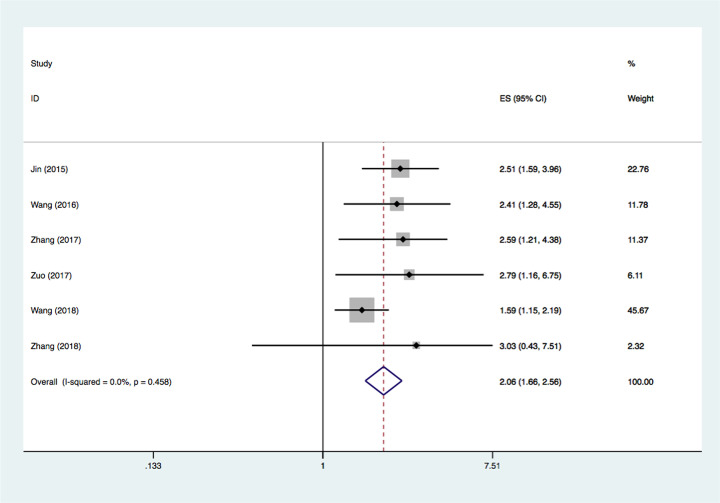
Forest plots of studies evaluating the association between MALAT-1 expression and OS in patients with breast cancer

Six studies evaluated the connection between MALAT-1 expression levels and OS [16,19,22,27,28,30]; no statistically significant heterogeneity was observed among the six studies (*I^2^* = 0.0%, *P*=0.458). Thus, the fixed-effects model was applied. Collectively, the results indicated that increased MALAT-1 expression in breast cancer was related to a worse OS (HR = 2.06, 95% CI: 1.66–2.56, *P*<0.0001) (Figure 2). Next, we performed subgroup analysis according to cancer type, sample size, and the method of variance analysis. As shown in Table 2, the subgroup analysis for cancer type suggested that MALAT-1 up-regulation was a strong prognostic biomarker in both patients with TNBC (HR = 1.88, 95% CI = 1.45‒2.44, *P*<0.0001) and in those with mixed type breast cancer (HR = 2.57, 95% CI = 1.71‒3.85, *P*<0.0001). Next, we performed subgroup analysis according to the sample size, with a threshold for 100 patients. The results showed that the association of MALAT-1 dysregulation and OS was not significantly influenced by sample size (*n*≥100: HR = 2.00, 95% CI = 1.59‒2.51, *P*<0.0001; *n*<100: HR = 2.86, 95% CI = 1.35‒6.04, *P*=0.006). Similarly, pooled results of subgroup analysis according to the analysis method revealed that elevated MALAT-1 expression was significantly associated with worse OS in both the multivariate (HR = 1.75, 95% CI = 1.31‒2.34, *P*<0.0001) and univariate groups (HR = 2.55, 95% CI = 1.83‒3.55, *P*<0.0001).

**Table 2:** Results of subgroup analyses of pooled HRs for OS with elevated MALAT-1 expression

Categories	Number of studies	Number of patients	Pooled HR (95% CI)	Heterogeneity	
				*I^2^* (%)	*P*-value
Cancer type					
Mixed	4	891	2.57 (1.71, 3.85)	0.4	0.834
TNBC	2	182	1.88 (1.45, 2.44)	0	0.39
Sample size					
≥100	4	944	2.00 (1.59, 2.51)	22.2	0.277
<100	2	128	2.85 (1.35, 6.04)	0	0.923
Analysis method					
Univariate	4	386	2.55 (1.83, 3.55)	0	0.988
Multivariate	2	687	1.75 (1.31, 2.34)	43.4	0.184

### Association between MALAT-1 expression and DFS/RFS/DSS

**Figure 3:**
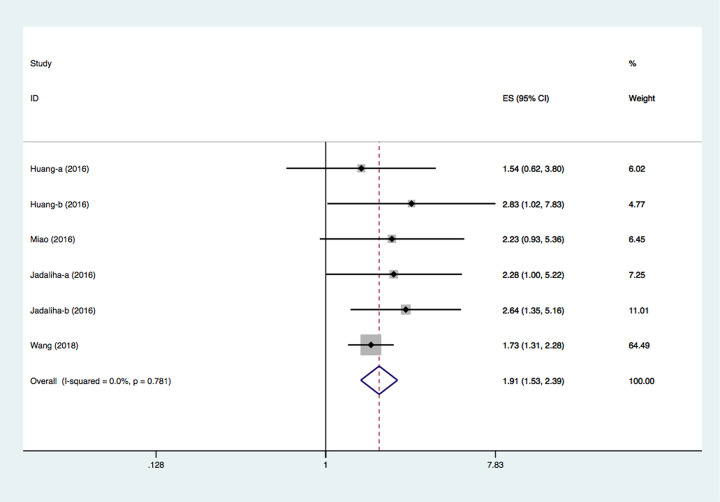
Forest plots of studies evaluating the association between MALAT-1 expression and DFS/RFS/DSS in patients with breast cancer

Six studies investigated the connection between MALAT-1 expression levels and DFS/MFS/DSS/RFS [19,23–26,29]. Li et al.’s study suggested that low MALAT-1 expression predicted favorable RFS in their cohort (HR = 0.0093, 95% CI: 0.0013‒0.0637, *P*<0.001) [29]. Their data were insufficient for quantitative analysis but were still included in the qualitative analysis. Meseure et al.’s study suggested that the impact of MALAT-1 expression level on MFS was not significant. Unfortunately, this study did not provide useful data for HR calculation [25]. Eventually, a total of four studies compromising six groups of data reported the prognostic significance of MALAT-1 for DFS, RFS, and DSS. The pooled HR was 1.91 (95% CI: 1.53–2.39, *P*<0.0001, using the fixed-effects model) (Figure 3). On account of the limited number of studies, a subgroup analysis for DFS/RFS/DSS was not performed.

### Association between MALAT-1 expression and clinicopathological parameters

We also evaluated the relationship between the expression level of MALAT-1 and the clinicopathological features by pooling the clinicopathological data. As shown in Table 3, we found that high expression of MALAT-1 was significantly associated with PR status (OR = 1.47, 95% CI = 1.18–1.82, *P*=0.0006, fixed-effects model). Unlike the PR status, the ER status did not display a significant correlation with MALAT-1 expression, as there was great heterogeneity (OR = 1.37, 95% CI = 0.85‒2.22, *P*=0.19, random-effects model). In addition, a significant correlation was not found at TNM stage (OR = 1.27, 95% CI = 0.56‒2.87, *P*=0.57, random-effects model), tumor size (OR = 0.95, 95% CI = 0.69–1.31, *P*=0.75, fixed-effects model), distant metastasis (OR = 1.17, 95% CI = 0.27– 5.15, *P*=0.83, random-effects model), lymph node metastasis (OR = 0.86, 95% CI = 0.41– 1.82, *P*=0.7, random-effects model), histological grade (OR = 0.90, 95% CI = 0.47–1.71, *P*=0.74, random-effects model), and HER2 status (OR = 1.10, 95% CI = 0.81–1.48, *P*=0.56, fixed-effects model).

**Table 3:** Meta-analyses of MALAT-1 expression with respect to clinicopathologic parameters

Variables	Number of studies	Number of patients	Model	OR (95% CI)	*P-*value	Heterogeneity (*I^2^, P*-value)
TNM stage (III/IV vs. I/II)	5	902	Random	1.27 (0.56, 2.87)	0.57	82%, 0.0002
Tumor size (>2 vs <2 cm)	6	639	Fixed	0.95 (0.69, 1.31)	0.75	0.0%, 0.58
Distant metastasis (positive vs. negative)	4	386	Random	1.17 (0.27, 5.15)	0.83	89%, <0.00001
Lymph node metastasis (positive vs. negative)	4	862	Random	0.86 (0.41, 1.82)	0.7	77%, 0.004
Histological grade (III vs. I/II)	5	937	Random	0.90 (0.47, 1.71)	0.74	74%, 0.004
ER status (positive vs. negative)	8	1380	Random	1.38 (0.85, 2.22)	0.19	72.0%, 0.0008
PR status (positive vs. negative)	8	1400	Fixed	1.47 (1.18, 1.82)	0.0006	0.0%, 0.44
HER2 status (positive vs. negative)	6	1007	Fixed	1.10 (0.81, 1.48)	0.56	0.0%, 0.84

### Sensitivity analysis and publication bias

**Figure 4:**
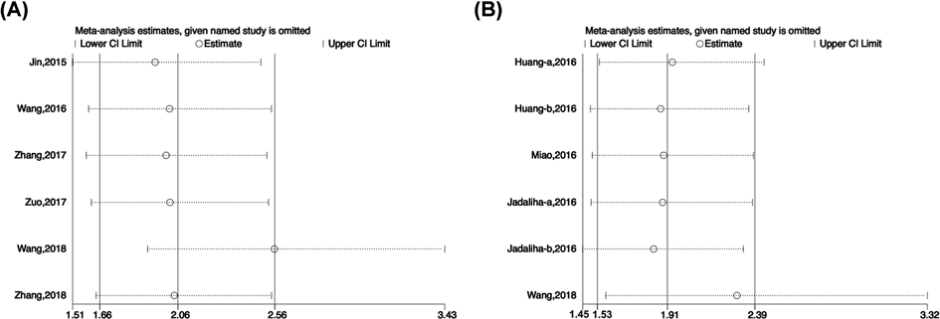
Sensitivity analysis of the pooled HRs of MALAT-1 expression (**A**) OS; (**B**) DFS/RFS/DSS.

We performed sensitivity analysis to examine the stability of the meta-analysis. When any one study was sequentially omitted, no individual study dominantly affected the overall HR for OS (Figure 4A) or DFS, RFS, or DSS (Figure 4B).

**Figure 5:**
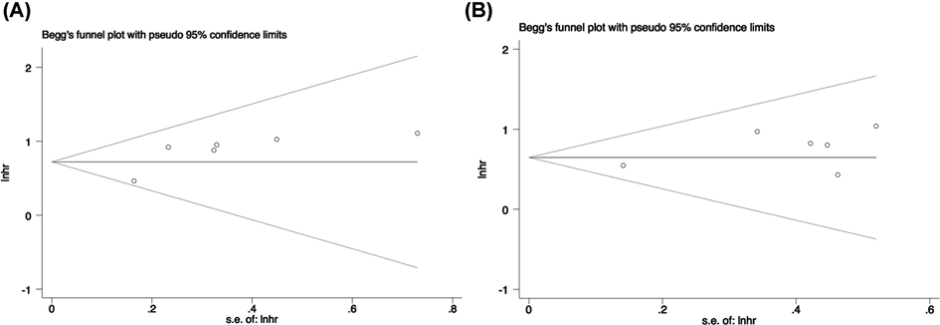
Funnel plot of publication bias analysis (**A**) OS; (**B**) DFS/RFS/DSS.

Egger’s test and Begg’s funnel plot were used to test publication bias. As shown in Figure 5A,B, the funnel plot demonstrated basic symmetrical shape in OS studies (Egger’s test *P*=0.071) and in DFS/RFS/DSS studies (Egger’s test *P*=0.142). Hence, there was no noticeable publication bias in the current meta-analysis.

### Breast cancer patient cohort analysis based on public datasets

We also checked MALAT-1 expression levels in chemotherapy-treated patients based on publicly available microarray datasets. Our analysis showed that the expression level of MALAT-1 was markedly lower in the chemotherapy-treated groups than that in the non-chemotherapy group (Supplementary Figure S1). Indeed, MALAT-1 could not predict prognosis in these groups (data not shown). These findings suggested that the prognostic value of MALAT-1 for patients receiving chemotherapy requires further investigation.

**Figure 6:**
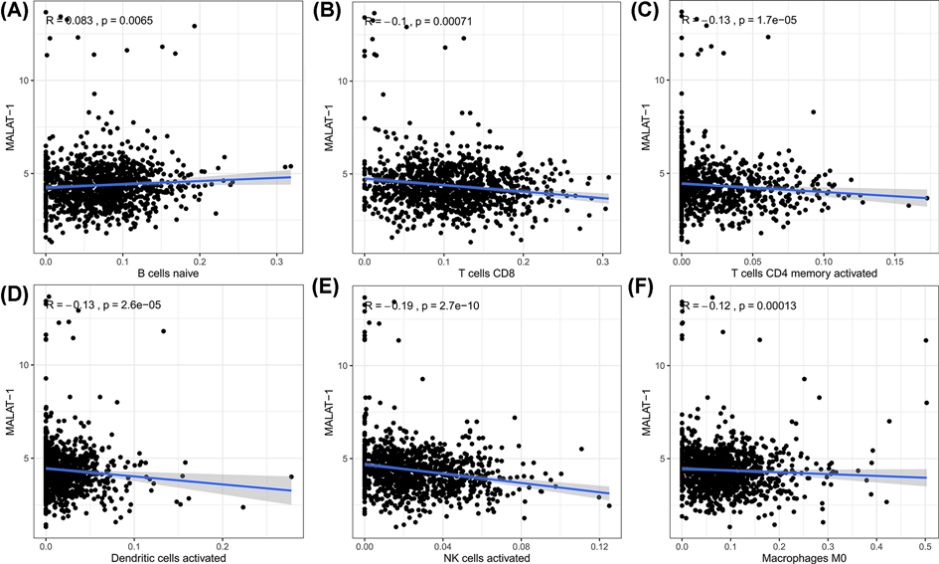
Correlation between MALAT-1 and infiltrating immune cells (**A**) association between MALAT-1 and B cells; (**B**) association between MALAT-1 and CD8^+^ T cells; (**C**) association between MALAT-1 and CD4^+^ T cells; (**D**) association between MALAT-1 and dendritic cells; (**E**) association between MALAT-1 and NK cells; (**F**) association between MALAT-1 and macrophages M0.

To further explore the possible reason why MALAT-1 is responsible for poor survival in patients with breast cancer, we analyzed the association between MALAT-1 and tumor infiltrating immune cells based on TCGA dataset. As shown in Figure 6, MALAT-1 expression levels showed a significant positive correlation with immune infiltration by B cells (*R* = 0.083, *P*=0.0065) and a negative association with CD8^+^ T cells (*R* = −0.1, *P*=0.00071), CD4^+^ T cells (*R* = −0.13, *P*=1.7e−05), dendritic cells (*R* = −0.13, *P*<2.6e−05), NK cells (*R* = −0.19, *P*=2.7e−10), and macrophages M0 (*R* = −0.12, *P*=0.00013). These findings strongly implied that MALAT-1 may be implicated in immune infiltration in patients with breast cancer.

## Discussion

MALAT-1, also referred to as noncoding nuclear-enriched abundant transcript 2, was initially identified to promote metastasis in non-small-cell lung cancer [13,14]. To date, aberrant expression of MALAT-1 has been found in multiple cancers, such as ovarian, breast, colorectal, and bladder cancers [19,31–33]. Some studies have shown that elevated MALAT-1 expression levels contribute to breast cancer carcinogenesis, whereas a few studies have demonstrated that MALAT-1 may serve as a tumor-suppressing gene [34,35]. Hence, the role of MALAT-1 and its influence on survival outcomes in patients with breast cancer remain controversial. In addition, although previously published reviews and meta-analyses have reported that MALAT-1 could function as a potential prognostic biomarker in cancers, no studies have focused on its prognostic significance in breast cancer. Here, we pooled published data to highlight the prognostic and clinical value of MALAT-1 in breast cancer. It is well-known that breast cancer is a highly heterogeneous disease. Based on the heterogeneity of ER and HER2 expression, breast cancer can be classified into three main subtypes: luminal (luminal A and luminal B), HER2-positive, and TNBC. These subtypes display distinct histological features, molecular etiologies, and clinical behaviors [5,6]. Our comprehensive meta-analysis of 4186 cases from 12 cohorts showed that high MALAT-1 expression levels in patients with breast cancer were observed in most studies, with no obvious subtype or cell specificity. Patients with breast cancer having elevated MALAT-1 expression levels displayed worse survival outcomes (both OS and DFS/RFS/DSS). The association between MALAT-1 and poor prognosis in patients with breast cancer was consistent in most of the original studies as well as in the reprocessed data obtained from TCGA database [36]. The biological behavior of MALAT-1 in cancer may explain this correlation. First, MALAT-1 is involved in pre-mRNA alternative splicing through interactions with serine- and arginine-rich proteins [37,38]. Second, MALAT-1 participates in transcriptional regulation. MALAT-1 actively interacts with the 3′ end of the gene body and overlaps with H3K36me2 peaks, a marker of active transcriptional elongation, indicating its role in gene expression [13]. Third, MALAT-1 can function as a post-transcriptional regulator of gene expression through a ‘ceRNA’ mechanism. MALAT-1 utilizes miRNA-responsive elements in miRNA sequences as a ‘language’ to communicate with mRNAs and pseudogenes, thereby resulting in phenotypic alterations such as cell invasion and metastasis [16,39]. Multiple studies have suggested that MALAT-1 plays oncogenic roles in breast cancer. For instance, MALAT-1 modulates cdc42 expression by sponging miR-1 in cell lines, thereby triggering migration and invasion [40]. MALAT-1 can promote angiogenesis by interacting with miR-145 [41]. MALAT-1 also contributes to the maintenance of stem cell-like phenotypes in breast cancer cells by regulating self-renewal-associated factors [42]. Moreover, MALAT-1 exerts a crucial role in tumor progression and metastasis in both TNBC and luminal cells [23,28]. Immune cell infiltration and the tumor microenvironment have been verified to play essential roles in the initiation and progression of cancers [43,44]. Low levels of immune-infiltrating cells in the tumor microenvironment may confer worse prognosis in breast cancer [45,46]. In our study, high MALAT-1 expression levels were associated with low immune cell infiltration (e.g., CD4^+^ and CD8^+^ T cells), which may explain the correlation between MALAT-1 and poor prognosis in patients with breast cancer. Taken together, the extensive range of functions of MALAT-1 enables it to predict survival outcomes in patients with breast cancer.

Notably, our results suggested that high MALAT-1 expression levels were significantly associated with PR status (OR = 1.47, 95% CI = 1.18–1.82). ER status also had a tendency for correlation with MALAT-1 expression, albeit not significant (*P*>0.05) due to the evident heterogeneity. Previous studies have shown that MALAT-1 regulates the effect of 17β-estradiol treatment on breast cell lines [47] and is associated with ER and its target genes. Moreover, MALAT-1 might confer tamoxifen resistance by affecting the ER signaling pathway [23,48]. These studies demonstrate that MALAT-1 is closely related to hormone receptor status in breast cancer, which is consistent with our results. No significant association was observed between MALAT-1 expression and advanced clinical parameters, such as distant metastasis, lymph node metastasis, and TNM stage. This negative result seems reasonable, given the limited number of enrolled patients as well as the diverse enrollment criteria among studies. In addition, Kim et al. [34] and Kwok et al. [35] recently demonstrated a metastasis-suppressing rather than a metastasis-promoting role of MALAT-1 in breast cancer. Moreover, multiple molecular networks, and not MALAT-1 alone, contribute to tumor development. Therefore, further studies should be conducted to identify the function of MALAT-1 in breast cancer and verify the association between clinical parameters and MALAT-1 expression.

Although the prognostic value of MALAT-1 was identified in this meta-analysis, there are a few limitations of the study. First, marked heterogeneity existed owing to the difference in methodology, such as in the determination of cut-off values, sample selection, RT-PCR primer sets, and statistical analyses. We applied the random-effects model to minimize the influence of these differences when heterogeneity was large. Second, HR may have been overestimated because only two studies provided multivariate HR estimate for survival outcomes. In addition, the HR value extracted from the survival curve might cause slight error for prognosis. Third, we should carefully consider the preference for publications with significant results, as studies showing significant results are easier to publish than negative ones. Notably, Messeur et al. [25] did not report the negative result regarding MALAT-1 and breast cancer prognosis, whereas Jadaliha et al. [24] and Zuo et al. [28] only reported significant results for specific groups. Fourth, the sample size of some included studies was small (<100), and smaller studies could obtain higher effect size as well as heterogeneity compared with larger studies. Finally, language bias could also have been implicated as the selected studies were only published in English.

In conclusion, our study indicated that elevated MALAT-1 expression levels are predictive of unfavorable prognosis, not only for OS but also DFS, RFS, and DSS in breast cancer. Therefore, it could potentially function as a prognostic biomarker for patients with breast cancer. Furthermore, our results demonstrated that the level of MALAT-1 tended to correlate with hormone receptor status. Nevertheless, due to the limitations mentioned above, additional comprehensive, well-designed multicenter studies should be performed to confirm and strengthen our findings regarding the prognostic role of MALAT-1 in breast cancer.

## Supplementary Material

Supplementary Figure S1

## Abbreviations

ceRNA: competitive endogenous RNA
CI: confidence interval
DFS: disease-free survival
DSS: disease-specific survival
ER: estrogen receptor
HER2: human epidermal growth factor receptor 2
HR: hazard ratio
H3K36me2: Histone H3 dimethylation at lysine 36
lncRNA: long noncoding RNA
MALAT-1: metastasis-associated lung adenocarcinoma transcript 1
MFS: metastasis-free survival
NK: natural killer
NOS: Newcastle–Ottawa Scale
OR: odds ratio
OS: overall survival
PR: progesterone receptor
RFS: recurrence-free survival
RT-PCR: reverse transcriptase polymerase chain reaction
TCGA: The Cancer Genome Atlas
TNBC: triple-negative breast cancer
TNM: Tumor Node Metastasis
ZEB: zinc-finger E-box-binding homeobox
