# Supplementary material for: Elevated long noncoding RNA MALAT-1 expression is predictive of poor prognosis in patients with breast cancer: a meta-analysis
Source: Biosci Rep. 2020 Aug 11;40(8):BSR20200215. doi: 10.1042/BSR20200215 (PMC7419804; doi:10.1042/BSR20200215)
Supplement: Supplementary Figure S1 [file BSR-2020-0215_supp.pdf]

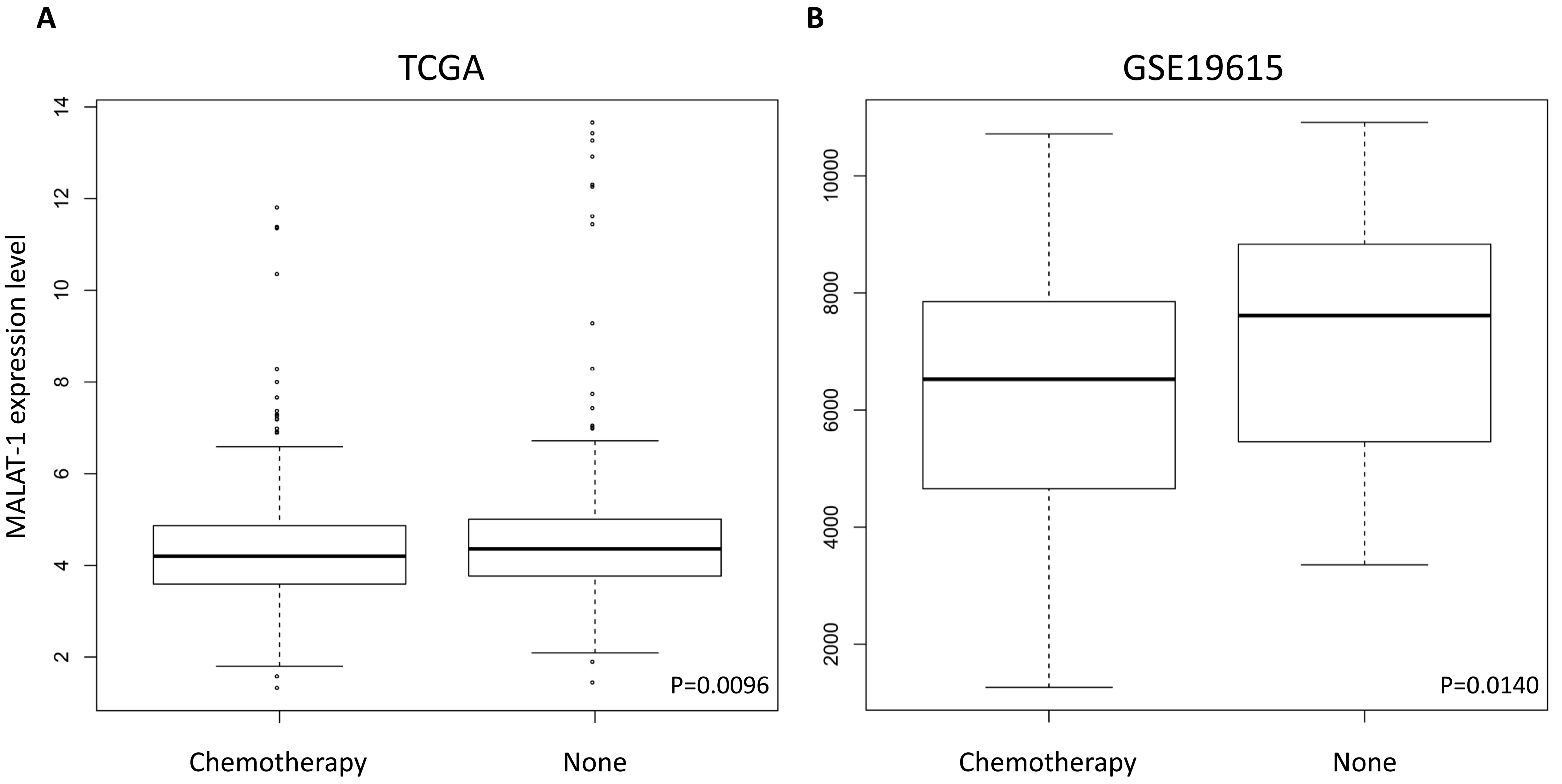
A
B
TCGA
GSE19615
MALAT-1 expression level
14
12
10
8
6
4
2
10000
8000
6000
4000
2000
P=0.0096
P=0.0140
Chemotherapy
None
Chemotherapy
None

Figure. S1. Box-plot data depicting the relative levels of MALAT-1 in breast cancer patient samples from public microarray datasets: (A) TCGA microarray dataset; (B) GSE19165 dataset from GEO
